# Heart rate responses induced by acoustic tempo and its interaction with basal heart rate

**DOI:** 10.1038/srep43856

**Published:** 2017-03-07

**Authors:** Ken Watanabe, Yuuki Ooishi, Makio Kashino

**Affiliations:** 1 Department of Information Processing, Interdisciplinary Graduate School of Science and Engineering, Tokyo Institute of Technology, 4259 Nagatsuta-cho, Midori-ku, Yokohama, Kanagawa 226-8503, Japan; 2 NTT Communication Science Laboratories, NTT Corporation, 3-1, Morinosato Wakamiya Atsugi, Kanagawa 243-0198, Japan; 3 Core Research for Evolutional Science and Technology, Japan Science and Technology Agency (CREST, JST), Atsugi, Kanagawa 243-0198, Japan

## Abstract

Many studies have revealed the influences of music on the autonomic nervous system (ANS). Since previous studies focused on the effects of acoustic tempo on the ANS, and humans have their own physiological oscillations such as the heart rate (HR), the effects of acoustic tempo might depend on the HR. Here we show the relationship between HR elevation induced by acoustic tempo and individual basal HR. Since high tempo-induced HR elevation requires fast respiration, which is based on sympatho-respiratory coupling, we controlled the participants’ respiration at a faster rate (20 CPM) than usual (15 CPM). We found that sound stimuli with a faster tempo than the individual basal HR increased the HR. However, the HR increased following a gradual increase in the acoustic tempo only when the extent of the gradual increase in tempo was within a specific range (around + 2%/min). The HR did not follow the increase in acoustic tempo when the rate of the increase in the acoustic tempo exceeded 3% per minute. These results suggest that the effect of the sympatho-respiratory coupling underlying the HR elevation caused by a high acoustic tempo depends on the basal HR, and the strength and the temporal dynamics of the tempo.

Many studies have revealed the influences of music on human beings and have adopted various ways of estimating these effects^1^,^2^,^3^,^4^. Particularly, music-induced emotion such as pleasure or happiness has been broadly investigated in brain imaging studies and physiological studies. The intense pleasure aroused in response to music can lead to dopamine release in the striatal system^5^. Such music-induced pleasure is also represented as an increase in frontal midline theta power^6^. Concerning music-induced relaxation, Okada *et al*. reported that music therapy induces an increase in parasympathetic nervous activity and a decrease in sympathetic nervous activity^7^.

Compared with the many studies demonstrating the relationship between music-induced emotion and brain functions or physiological responses, there has been relatively little work examining the effects of the acoustic characteristics of music (tempo, harmonic structure, rhythm or sound pressure level). Of these musical characteristics, it has been suggested that “tempo” plays a critical role for determining whether the effect of listening to music is exciting or relaxing^8^. Earlier studies reported that listening to music with a fast tempo caused an increase in the heart rate (HR) and the respiratory rate^8^,^9^.

However, these previous studies could not separate the effects of respiration on sympathetic nerve activity when a subject was listening to high tempo sound. The effects of respiration on the ANS should be considered because there is a neural connection between the respiratory system and the rostral ventrolateral medulla (RVLM), which is the primary regulator of the sympathetic nervous system concerned with blood pressure. This connection is known as sympatho-respiratory coupling and is closely related to the sympathetic tone induced by auditory stimuli^10^,^11^,^12^,^13^,^14^,^15^. Our previous study separately evaluated the effects of the respiratory system and the auditory system on the ANS by controlling both respiratory rate and acoustic tempo. We previously showed that an increase in HR induced by a high acoustic tempo requires fast respiration, suggesting that the increase in HR should be based on sympatho-respiratory coupling^16^.

Considering the fact that humans have their own oscillation, for example the HR, there is a possibility that the elevation of the HR by a high acoustic tempo is regulated by the HR itself rather than sympatho-respiratory coupling. However, no study has considered this. In this study, we investigated the contribution of the individual basal HR to the HR elevation induced by a high acoustic tempo with fast respiration.

## Results

### Experiment 1

#### Correlation between individual basal HR and ratio of change in mean HR when listening to high tempo sounds

**Figure 1:**
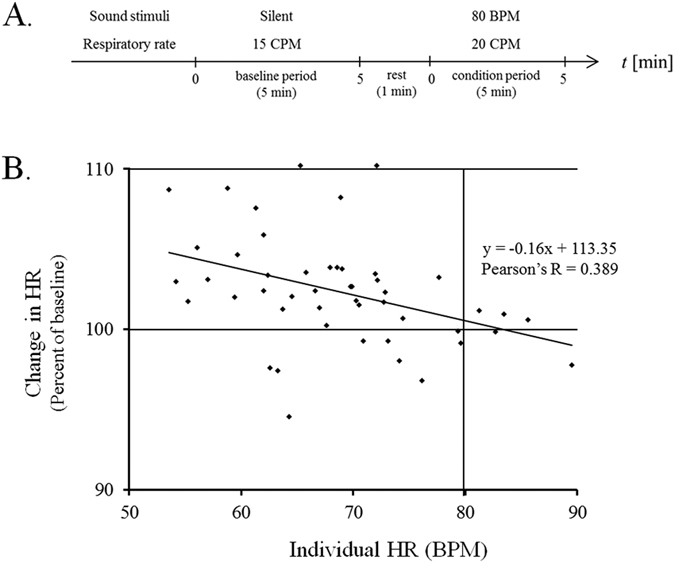
Correlation between individual HR and change in HR. (**A**) Experimental procedure for Experiment 1. The experimental procedure consisted of two periods and a 1-min rest: baseline period (5 min) → 1-min rest → condition period (5 min). (**B**) The horizontal axis indicates the individual HR, which is the average HR in baseline recording. The vertical axis indicates the change in HR, which is the average HR obtained during condition period recording. Each point represents the data set of one participant. Since the participants listened to the sound at 80 BPM in the condition period, the vertical line is drawn on the graph at 80 BPM. The diagonal line and equation indicate an approximately straight line and the approximation formula, respectively.

In Experiment 1, we investigated the correlation between the individual basal HR, which is obtained by averaging the HR in the baseline period, and the effects of sounds on the autonomic nervous system (ANS). We measured the HR to evaluate the change in ANS activity induced by simple drum sounds whose tempo was set at 80 beats per minute (BPM). Then, the mean HR was calculated by averaging the HR of the last three minutes in the listening session (condition period). The respiratory rates of participants were controlled at 20 cycles per minute (CPM) in the condition period (Fig. 1A). We compared the ratio of the change in the mean HR in the condition period with the individual basal HR in the baseline period. The experimental procedure for Experiment 1 is shown in Fig. 1A.

The results showed a significant negative correlation between the individual HR in the baseline period and the change in the mean HR in the condition period (Pearson’s R = 0.398, *p* = 0.0051). The approximation formula is represented by the equation described below.


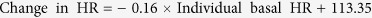


This negative correlation suggests that the modulation of the mean HR is in the direction from the individual HR at the baseline recording to the acoustic tempo (80 beats per minute (BPM)). The results of Experiment 1 are shown in Fig. 1B.

### Experiment 2

#### Effect of relationship between acoustic tempo and individual basal HR on ANS

**Figure 2:**
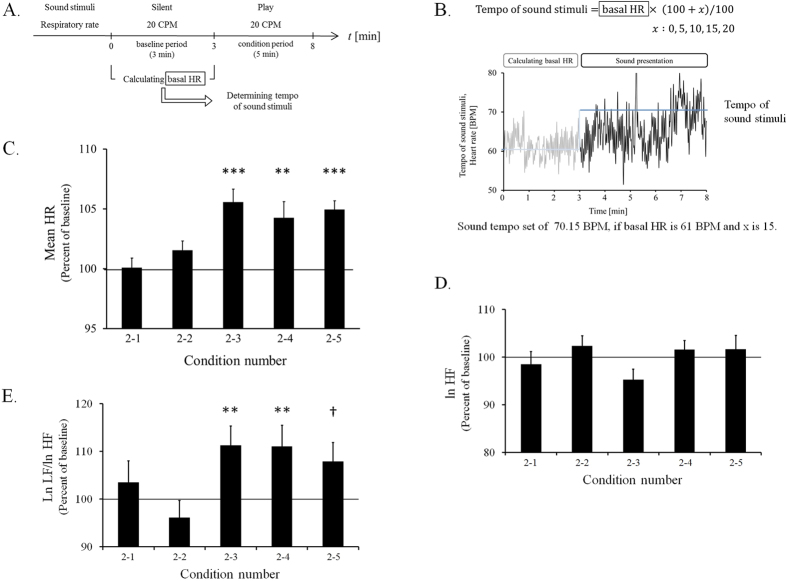
Normalized mean HR, lnHF and lnLF/HF of each condition in Experiment 2. (**A**) Procedure for Experiment 2. (**B**) The equation and an example of Experiment 2. The graph represents the time-series data of the HR of a participant. The solid blue line represents the tempo of sound in this condition (*x* = 15). (**C**) The vertical axis indicates the normalized mean HR (percent of baseline of each session). (**D**) The vertical axis indicates the normalized lnHF of the HRV (percent of baseline of each session). (**E**) The vertical axis indicates the normalized lnLF/lnHF ratio of the HRV (percent of baseline of each session). The horizontal axis indicates the condition number shown in Table 2. The acoustic tempo increases from left to right. The bar graphs and error bars represent the mean ± SEM. Statistical significance is indicated as *p < 0.05, **p < 0.01, ***p < 0.001 from the baseline. The trend of the increase is indicated as ^†^*p* < 0.1 compared with the baseline.

From the results of Experiment 1, there is a negative correlation between the individual basal HR in the resting state and the change in the HR when listening to high-tempo sound. Therefore, we presented sound at a faster tempo than the individual basal HR to examine the relationship between acoustic tempo and individual basal HR (Fig. 2). We evaluated the effect of the relationship between acoustic tempo and individual basal HR on autonomic nerve activity by measuring the change in the mean HR (Fig. 2A). A faster tempo sound whose tempo was determined with reference to the individual basal HR (Fig. 2B) induced a significant increase in the mean HR and this increase appears to become saturated (Fig. 2C).

The mean HR for each condition was normalized by the basal HR in the baseline recording in each session. The percentages and SEMs of the normalized HR were 100.10 ± 0.79%, 101.55 ± 0.77%, 105.58 ± 1.07%, 104.26 ± 1.33% and 104.94 ± 0.74% under conditions 2–1, 2–2, 2–3, 2–4 and 2–5, respectively. The two-way repeated measures analysis of variance (ANOVA) yielded the condition × time interaction, *F*(4, 56) = 7.847, *p* = 0.000043, a main effect of condition, *F*(4, 56) = 7.847, *p* = 0.000043, and a main effect of time, *F*(1, 14) = 28.870, *p* = 0.000097. A simple main effect test demonstrated that there was a significant difference between the normalized mean HR and the basal HR in a baseline recording under conditions 2–3, 2–4, and 2–5. The normalized mean HR was significantly larger than the basal HR under conditions 2–3 (*F*(1, 70) = 33.185, *p* = 2.1 × 10^−7^), 2–4 (*F*(1, 70) = 19.385, *p* = 3.8 × 10^−5^) and 2–5 (*F*(1, 70) = 26.052, *p* = 2.7 × 10^−6^). A simple main effect test followed by Ryan’s method of adjusting the P value demonstrated that there was a significant difference between the normalized mean HR under some conditions (*F*(4, 112) = 15.693, *p* = 3.2 × 10^−10^). The normalized mean HR was significantly larger for condition 2–3 than for conditions 2–1 (*p* = 2.0 × 10^−8^) and 2–2 (*p* = 3.8 × 10^−5^), for condition 2–4 than for conditions 2–1 (*p* = 1.3 × 10^−5^) and 2–2 (*p* = 0.0041), and for condition 2–5 than for conditions 2–1 (*p* = 5.4 × 10^−7^) and 2–2 (*p* = 0.00049).

The procedure and the normalized mean HRs of each condition in Experiment 2 are shown in Fig. 2.

Then we evaluated the parasympathetic influence on the heart by calculating the natural logarithms of the power of the high frequency component (lnHF) of the heart rate variability (HRV). The lnHF was averaged with the two 2-min windows and normalized by the baseline recording value in each session. The percentages and SEMs of the normalized lnHF of the HRV were 98.51 ± 2.63%, 102.31 ± 2.14%, 95.26 ± 2.18%, 101.53 ± 1.90% and 101.65 ± 2.89% under conditions 2–1, 2–2, 2–3, 2–4 and 2–5, respectively (Fig. 2D). However, we could not observe any significant difference in the lnHFs with the five conditions and times.

We evaluated the sympathetic influence on the heart by calculating the ratio of the natural logarithms of the power of the low frequency component (lnLF) to the lnHF of the HRV. The lnLF/lnHF ratio was averaged with the two 2-min windows and normalized by the baseline recording value in each session. The percentages and SEMs of the normalized lnLF/lnHF ratio of the HRV were 103.46 ± 4.55%, 96.11 ± 3.58%, 111.22 ± 4.09%, 110.98 ± 4.48% and 107.88 ± 3.94% under conditions 2–1, 2–2, 2–3, 2–4 and 2–5, respectively (Fig. 2E). A two-way repeated measures ANOVA yielded the condition × time interaction, *F*(4, 56) = 2.922, *p* = 0.029, a main effect of condition, *F*(4, 56) = 2.922, *p* = 0.029, and a main effect of time, *F*(1, 14) = 5.659, *p* = 0.032. A simple main effect test demonstrated that there was a significant difference between the normalized lnLF/lnHF ratio and the basal lnLF/lnHF ratio in a baseline recording under conditions 2–3 and 2–4. The normalized lnLF/lnHF ratio was significantly larger than the basal lnLF/lnHF ratio under conditions 2–3 (*F*(1, 70) = 7.334, *p* = 0.0085) and 2–4 (*F*(1, 70) = 7.030, *p* = 0.0099). A simple main effect test revealed a tendency for the lnLF/lnHF ratio to increase compared with the baseline under condition 2–5 (*F*(1, 70) = 3.618, *p* = 0.061). A simple main effect test followed by Ryan’s method of adjusting the P value demonstrated that there was a significant difference between the normalized mean HR under some conditions (*F*(4, 112) = 5.843, *p* = 0.0003). The normalized lnLF/lnHF ratio was significantly larger for condition 2–3 than for condition 2–2 (*p* = 0.00083), for condition 2–4 than for condition 2–2 (*p* = 0.00079), and for condition 2–5 than for condition 2–2 (*p* = 0.0094).

The normalized lnHF and lnLF/lnHF ratio of the HRV for each condition in Experiment 2 are shown in Fig. 2. The basal value of HR, lnHF and lnLF/lnHF in the baseline recording for each condition in Experiment 2 are shown in Supplementary Table S1.

### Experiment 3

#### Effect of temporal drift of acoustic tempo from individual basal HR on ANS

**Figure 3:**
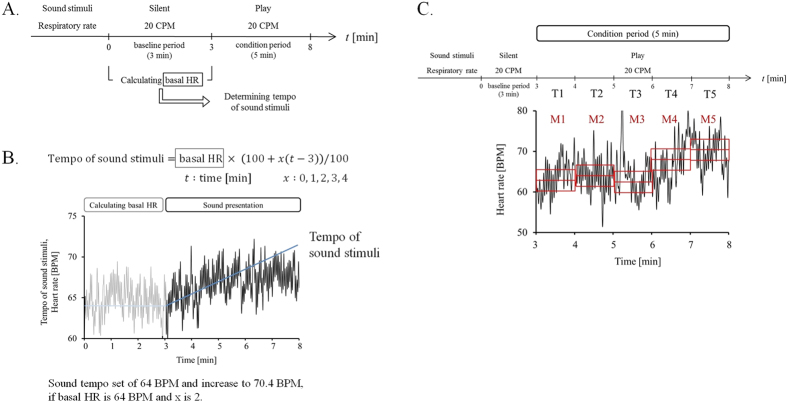
Time series of average HRs of all participants in Experiment 3. (**A**) Procedure for Experiment 2. (**B**) The equation and an example of Experiment 2. The graph represents the time-series data of the HR of a participant. The solid blue line represents the tempo of sound in this condition (*x* = 2). (**C**) The definitions of M1-M5, and T1-T5. The mean HRs of the 1-min windows were defined as M1, M2, M3, M4 and M5 in 5-min condition periods, and the corresponding 1-min time regions were defined as T1, T2, T3, T4, and T5, respectively.

From results of Experiments 1 and 2, the change in the HR is in the direction from the individual basal HR to the acoustic tempo when the tempo is higher than the basal HR. Since we showed the significant effects of acoustic tempo on the HR only when the tempo of sound is constant under these conditions, we investigated whether or not the change in the HR approaching the acoustic tempo occurs by presenting sound stimuli when the acoustic tempo varies with time (Fig. 3). Then, we presented sound stimuli with a gradually increasing tempo to confirm whether or not the HR follows the temporal drift of the acoustic tempo (Fig. 3A,B).

**Figure 4:**
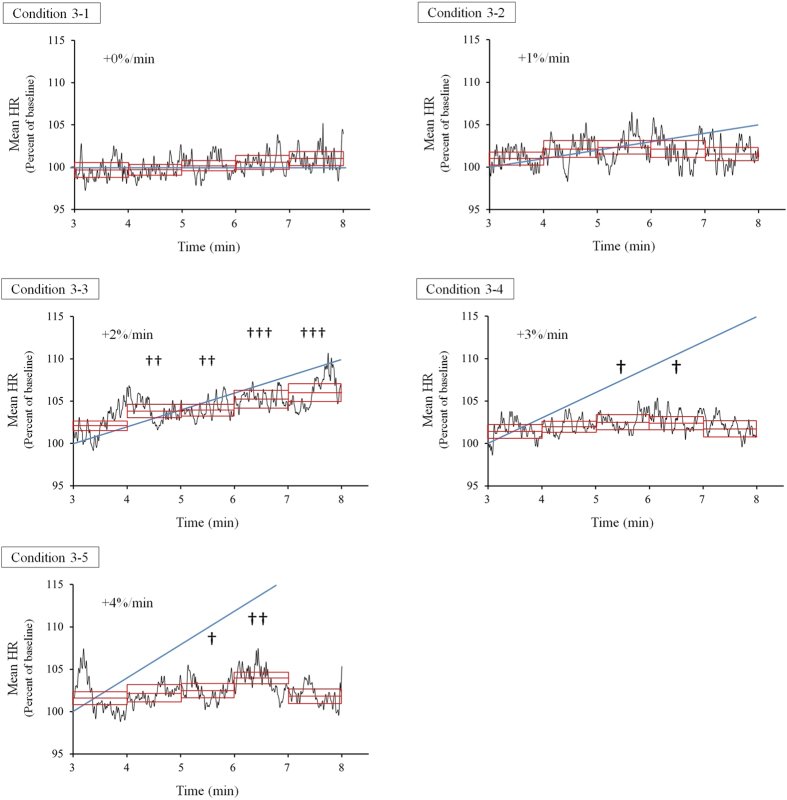
Time series of average HRs of all participants in Experiment 3. The data are normalized by the basal HR in the baseline recording. Each graph represents the time series HRs under each condition shown in Table 2. The vertical axis indicates the normalized mean HR (percent of baseline of each session). The horizontal axis indicates the time after the presentation of the sound stimuli. The rate of increase of the acoustic tempo per minute is indicated in each graph. The blue solid line represents the sound tempo normalized by the start of the acoustic tempo. The red box represents the mean value (center line) and SEM (top and bottom line). Statistical significance is indicated as ^†^*p* < 0.05, ^††^*p* < 0.01, ^†††^*p* < 0.001 from the baseline.

The effect of the time variation of the acoustic tempo on autonomic nervous activity was evaluated by measuring the change in the time variation of the mean HR. We observed that the mean HR increased following a gradual increase in the acoustic tempo only when that gradual increase was 2% per minute, which we named condition 3–3 (Fig. 4).

The average HR with a 1-min window (M1, M2, M3, M4 and M5, see Fig. 3C) for each condition was normalized by the basal HR of the baseline recording in each session. The percentages and SEM of the normalized HR are shown in Table 1. The two-way repeated measures ANOVA yielded a condition × time interaction, *F*(20, 400) = 2.007, *p* = 0.0065, a main effect of condition, *F*(4, 80) = 6.096, *p* = 0.00025, and a main effect of time, *F*(5, 100) = 14.739, *p* = 8.1 × 10^−11^.

A simple main effect test followed by Dunnett’s method revealed that there was a significant difference between the normalized mean HR and basal HR in baseline recording under conditions 3–3 (*F*(5, 500) = 13.258, *p* = 3.8 × 10^−12^), 3–4 (*F*(5, 500) = 2.388, *p* = 0.037) and 3–5 (*F*(5, 500) = 4.662, *p* = 0.00036). The normalized mean HR was significantly larger for M2 (*p* = 0.0013), M3 (*p* = 0.0011), M4 (*p* = 0.000021) and M5 (*p* = 8.6 × 10^−7^), as defined in Fig. 3C, than the basal HR under condition 3–3. The normalized mean HR was significantly larger for M3 (*p* = 0.019) and M4 (*p* = 0.024) than the basal HR under condition 3–4. The normalized mean HR was significantly larger for M3 (*p* = 0.023) and M4 (*p* = 0.00048) than the basal HR under condition 3–5. These statistical results are shown in Fig. 4 with dagger symbols.

**Figure 5:**
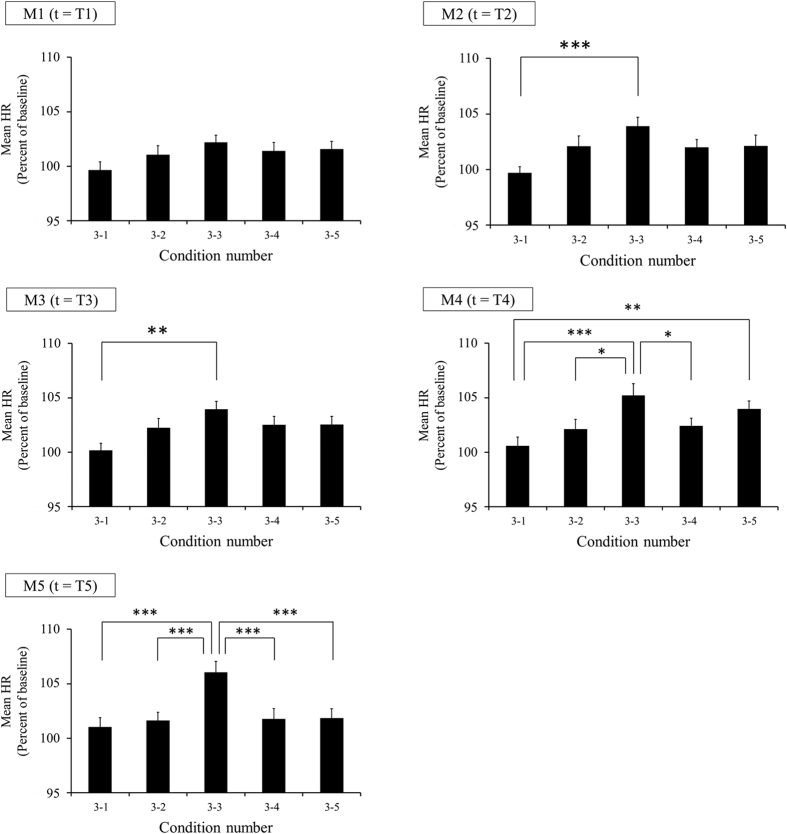
Mean HRs of the 1-min windows in Experiment 3. The data are normalized by the basal HR in the baseline recording. Each graph represents the mean HRs of the 1-min windows shown in Fig. 3C. The vertical axis indicates the normalized mean HR (percent of baseline of each session). The horizontal axis indicates the condition number shown in Table 2. The rate of increase in the acoustic tempo per minute is 0%/min, 1%/min, 2%/min, 3%/min and 4%/min, from left to right, respectively. The bar graphs and error bars represent the mean ± SEM. Statistical significance is indicated as *p < 0.05, **p < 0.01, ***p < 0.001.

A simple main effect test followed by Ryan’s method of adjusting the P-value showed that there was a significant difference between the normalized mean HRs under some conditions (M2: *F*(4, 480) = 4.155, *p* = 0.0026, M3: *F*(4, 480) = 3.461, *p* = 0.0084, M4: *F*(4, 480) = 5.891, *p* = 0.00012, M5: *F*(4, 480) = 7.741, *p* = 4.7 × 10^−6^). The normalized mean HR of M2 was significantly larger for condition 3–3 than for condition 3–1 (*p* = 0.00058). The normalized mean HR of M3 was significantly larger for condition 3–3 than for condition 3–1 (*p* = 0.0028). The normalized mean HR of M4 was significantly larger for condition 3–3 than for condition 3–1 (*p* = 0.0001), 3–2 (*p* = 0.022) and 3–4 (*p* = 0.037), and for condition 3–5 than for condition 3–1 (*p* = 0.009). The normalized mean HR of M5 was significantly larger for condition 3–3 than for condition 3–1 (*p* = 0.000016), 3–2 (*p* = 0.00016), 3–4 (*p* = 0.0002), and 3–5 (*p* = 0.00013). These statistical results are shown in Fig. 5 with asterisks.

The time series data of the normalized HR under each condition are shown in Fig. 4. These data were the average HRs for all the participants. The normalized mean HRs of the 1-min windows (T1, T2, T3, T4 and T5) are shown in Fig. 5.

**Figure 6:**
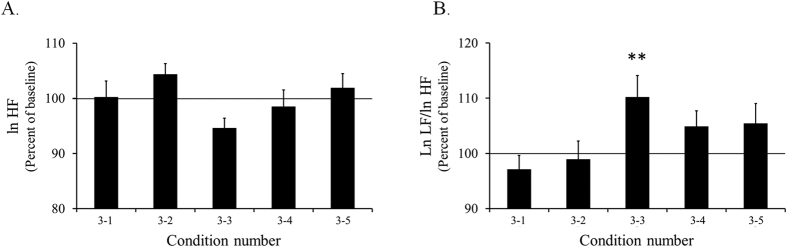
Normalized lnHF and lnLF/lnHF of HRV of each condition in Experiment 3. The horizontal axis indicates the condition number shown in Table 2. The acoustic tempo increases from left to right. (**A**) The vertical axis indicates the normalized lnHF of the HRV (percent of baseline of each session). (**B**) The vertical axis indicates the normalized lnLF/lnHF ratio of the HRV (percent of baseline of each session). The bar graphs and error bars represent the mean ± SEM. Statistical significance is indicated as **p < 0.01 from the baseline.

We then evaluated the parasympathetic influence on the heart by calculating the lnHF of the HRV. The lnHF was averaged with two 2-min windows and normalized by the baseline recording value in each session. The percentages and SEMs of the normalized lnHF of the HRV were 100.22 ± 2.91%, 104.34 ± 1.97%, 94.60 ± 1.81%, 98.48 ± 3.04% and 101.89 ± 2.58% under conditions 3–1, 3–2, 3–3, 3–4 and 3–5, respectively (Fig. 6A). However, we could not observe any significant difference in the lnHFs for the five conditions and times.

We evaluated the sympathetic influence on the heart by calculating the lnLF/lnHF ratio of the HRV. The lnLF/lnHF ratio was averaged with the two 2-min windows and normalized by the baseline recording value in each session. The percentages and SEMs of the normalized lnLF/lnHF of the HRV were 97.11 ± 2.55%, 98.90 ± 3.36%, 110.17 ± 3.96%, 104.90 ± 2.81% and 105.41 ± 3.63% under conditions 3–1, 3–2, 3–3, 3–4 and 3–5, respectively (Fig. 6B). A two-way repeated measures ANOVA yielded the condition × time interaction, *F*(4, 80) = 2.683, *p* = 0.037, a main effect of condition, *F*(4, 80) = 2.683, *p* = 0.037, and a main effect of time, *F*(1, 20) = 4.260, *p* = 0.052.

A simple main effect test demonstrated that there was a significant difference between the normalized lnLF/lnHF ratio and basal lnLF/lnHF ratio in a baseline recording under condition 3–3. The normalized lnLF/lnHF ratio was significantly larger than the basal lnLF/lnHF ratio under condition 3–3 (*F*(1, 100) = 9.500, *p* = 0.0027). A simple main effect test followed by Ryan’s method of adjusting the P value demonstrated that there was a significant difference between the normalized mean HR under some conditions (*F*(4, 160) = 5.366, *p* = 0.0004). The normalized lnLF/lnHF ratio was significantly larger for condition 3–3 than for condition 3–1 (*p* = 0.00081) and for condition 3–2 (*p* = 0.0047).

The normalized lnHF and lnLF/lnHF ratio of the HRV for each condition in Experiment 3 are shown in Fig. 6. The basal value of HR, lnHF and lnLF/lnHF in the baseline recording for each condition in Experiment 3 are shown in Supplementary Table S2.

## Discussion

The current study showed that individual physiological characteristics modulate the effect of sound stimuli on human beings. In particular, we observed that the HR appeared to follow the acoustic tempo only when the extent of the gradual increase in tempo was within a specific range (+2%/min).

The results of Experiment 1 showed that the change in HR induced by listening to the sound stimuli depended on the individual basal HR in the resting state when the respiration rate was controlled at a higher level (20 CPM) than normal (15 CPM). There was a significant negative correlation between the individual basal HR and the ratio of the change in HR. For example, the mean HRs of some participants were increased considerably by sound stimuli with an acoustic tempo of 80 BPM if the basal HR was slower than 80 BPM. On the other hand, the mean HR of another participant remained nearly unchanged if the basal HR was approximately 80 BPM, suggesting that the modulation of the HR was in the direction from the individual basal HR to the acoustic tempo. Then, in Experiment 2 we investigated the relationship between acoustic tempo and individual basal HR. As a result, we found that sound stimuli with a faster tempo than the individual basal HR caused a significant increase in the mean HR (Fig. 2C).

These results might be related to the ANS. The rostral ventrolateral medulla (RVLM) is one of the primary regulators of the sympathetic nervous system as regards vasoconstriction and arterial pressure, indicating that it is the regulator of the sympathetic nerve that controls cardiac activity^17^. The RVLM receives direct or indirect neuronal inputs from the auditory and respiratory systems^15^,^18^,^19^,^20^,^21^,^22^,^23^. In our previous study, we demonstrated that the activation of the sympathetic nerve induced by a high acoustic tempo requires a faster respiratory rate (20 CPM) than normal (15 CPM). Our previous study provided schematic views of the neural connectivity of the auditory system, amygdala, respiratory system and RVLM. From these neural connections, we previously suggested that sympatho-respiratory coupling works as an “enhancer” of the sympathetic tone when we listen to high-tempo sound stimuli^16^. Other studies indicated that an increase in RVLM activity is correlated with an increase in HR^24^,^25^. Taken together including the results of Experiment 2, we suggested that the effects of sound stimuli on the ANS depend on the RVLM activity in the resting state. For example, the RVLM activity is enhanced by sound stimuli with a low tempo if the basal RVLM activity is low. However, sound stimuli with a high tempo are needed for an increase in RVLM activity if the basal RVLM activity is high. Thus, the HR approached the acoustic tempo when the tempo of the sound stimuli was faster than the individual HR in baseline recording. We analyzed the lnHF and lnLF/lnHF ratio of the HRV to support this hypothesis. As a result, we observed a significant increase in the lnLF/lnHF ratio of the HRV, which is an index of sympathetic activity and is related to RVLM activity^26^, for conditions 2–3 and 2–4 compared with the baseline. For condition 2–5, we observed tendency for the lnLF/lHF ratio of the HRV to increase compared to baseline. On the other hand, we could not observe any significant difference between the lnHFs for the five conditions and times. We only observed an increase in the lnLF/lnHF ratio of the HRV when the mean HR was increased. Therefore, these data support the hypothesis described above.

However, the change in the HR in the acoustic tempo direction noted above is observed when the tempo of the sound is constant under these conditions. Then we investigated whether or not the HR followed an acoustic tempo that varied over time. As a result, we observed that the mean HR appeared to increase following a gradual increase in the acoustic tempo only when the extent of gradual increase in tempo was within a specific range (+2%/min). However, the mean HR did not increase with an increase in acoustic tempo but rather was close to the basal HR when the rate of increase in the acoustic tempo exceeded 3% per minute (Fig. 4).

These results suggested two possible mechanisms underlying the modulation of the ANS caused by listening to acoustic tempo. First, both enhancers and suppressors affect the HR, and the degree to which they influence the HR is balanced. Second, the activity of an enhancer is suppressed without any change in a suppressor. We analyzed the lnHF and the lnLF/lnHF ratio of the HRV to determine the more suitable hypothesis for explaining these results. As a result, we observed a significant increase in the lnLF/lnHF ratio of the HRV, which is an index of enhancer activity, for condition 3–3. For other conditions, we could not observe any significant difference in the lnLF/lnHF ratio (Fig. 6B). Moreover, we could not observe any significant difference in the lnHF, which is an index of suppressor activity^27^, for the five conditions and times (Fig. 6A). We only observed an increase in the lnLF/lnHF ratio of the HRV when the mean HR was gradually increased. Therefore, a gradual increase in the mean HR for condition 3–3 was caused by an increase in RVLM activity, similar to Experiments 1 and 2. On the other hand, the enhancement of sympathetic nerve activity, related to RVLM activity, may fail when the rate of increase in the acoustic tempo exceeds 3% per minute because we could not observe an increase in M5 and lnLF/lnHF ration of HRV for conditions 3–4 and 3–5. Taken together, these analyzed data supported the second hypothesis that the activity of an enhancer is suppressed without any change in a suppressor.

In this study we analyzed HRV to evaluate the autonomic nerve activity. However, some researchers have challenged the validity of the HRV and this problem is still under debate. The validity of the HF component of HRV is widely accepted as an index of the parasympathetic influence on the heart. In contrast, the validity of the LF/HF ratio of HRV as an index of cardiac control by sympathetic nerve activity or sympatho-vagal balance has been challenged by some studies^28^,^29^,^30^,^31^,^32^,^33^,^34^. Nevertheless, certain pharmacological evidence has influenced our study. Some studies have questioned the validity of the LF peak of HRV by dosing vagal blockade or beta-adrenergic blockade and reported that the LF peak of HRV could not reflect sympathetic nerve activity^33^,^34^,^35^,^36^. On the other hand, some studies have reported that a change in RVLM activity is consistently correlated with a change in HR and the LF/HF ratio of HRV. These studies measured HR, HRV and mean arterial pressure as an index of cardiac function when RVLM activity is affected by the microinjection of a pharmacological agent into the RVLM^26^,^37^,^38^,^39^,^40^. Therefore, we evaluated the LF/HF ratio of HRV as an index of cardiac sympathetic nerve activity derived from the RVLM. Moreover, the validity of the LF/HF ratio of HRV would not affect our main conclusion that the effect of acoustic tempo on HR is influenced by the relationship between acoustic tempo and basal HR.

Our study has one noteworthy limitation, namely that the respiratory rates of the participants should have been controlled at 20 CPM. Since the paced breathing influenced the HR and RSA, there is the possibility that we could not evaluate the cardiac vagal tone accurately in our experimental design^41^,^42^. However, when investigating the effects of acoustic tempo on HR we must maintain a fast respiration rate because our previous study showed that an increase in the HR induced by a high acoustic tempo requires fast respiration.

In this study, we discovered that the relationship between the tempo of sound stimuli and the individual basal HR in a resting state is important as regards changes in HR. In addition, sound stimuli with a gradual increase in tempo caused an increase in HR within a specific range. We suggest that the effect of the sympatho-respiratory coupling underlying the HR elevation induced by a high acoustic tempo depends on the basal HR, and the strength and the temporal dynamics of the tempo.

## Methods

### Ethics statement

Before the experiment, participants were provided with an information sheet that outlined the general purpose of the study and informed them that they could withdraw at any time without penalty. All methods employed in this study were approved by the Ethics and Safety Committees of NTT Communication Science Laboratories, and were in accordance with the Declaration of Helsinki. The protocol number of the Ethics and Safety Committees of NTT Communication Science Laboratories is H28–010.

### Participants

Ninety-six healthy people aged 20–40 years participated in the physiological experiments described below. The experiments were performed in a sound-insulated listening room. The participants sat on chairs and were encouraged to relax. The experiments were conducted between 13:00 and 18:00 h to standardize the circadian rhythms.

### Sound Stimuli & Respiration Regulation

We used 5-min sound sequences consisting of simple drum sounds. These stimuli were converted to analog signals with an audio interface (EDIROL UA-55, Roland, Japan) and presented through headphones. The sound pressure level (SPL) was determined by measuring the maximum A-weighted SPL of this sound in the slow mode. The participants listened to the sounds at 68 dB (A) in all the experiments and their respiratory rates were controlled by using a silent metronome displayed on a screen.

### Physiological Measurements

Electrocardiograms (ECGs) and an elastic chest band Polyam-RESP (Nihon Santeku, Japan) were used to measure interbeat intervals (R-R intervals) and respiration, respectively, throughout the experiments. The analog data were amplified and digitized with a BIOPAC MP150 (BIOPAC System, USA). The sampling rate was 1,000 Hz for all the measurements. We measured the respiration with the chest band alone to confirm the synchronization of the measured respiration with a metronome.

To calculate the R-R intervals in the ECG measurement, R-wave detection was performed with AcqKnowledge (analysis software produced by BIOPAC), and the result was visually screened to eliminate any inappropriate R-wave detection related to artifacts such as movement. The appropriately collected R-R interval data were resampled at 10 Hz by cubic spline interpolation. For HR analysis, these interpolated data of the R-R intervals were converted to second-by-second values and expressed as BPM by dividing 60 by each value.

We then calculated the HRV to estimate the magnitude of the ANS^43^. For the HRV analysis, we applied fast Fourier transformation (FFT) to this interpolated data of the R-R intervals. Prior to FFT, the linear trend was removed and a Hanning window was applied. The low-frequency (LF) and high-frequency (HF) components were obtained by integrating the power spectra over their respective ranges of 0.04–0.15 Hz and 0.15–0.40 Hz. The magnitudes of the LF and HF components were evaluated by using the natural logarithms of the LF and HF power (lnLF, lnHF respectively). The HF component of the HRV represents RSA, which is an index of parasympathetic activity^44^. The lnLF to lnHF component ratio (lnLF/lnHF ratio) was evaluated by dividing the lnLF component by the lnHF component. The lnLF/lnHF ratio of the HRV is an index of sympathetic nerve activity^45^,^46^.

### Experimental Procedure

The study consisted of three experiments, Experiment 1 (*n* = 50), Experiment 2 (*n* = 18) and Experiment 3 (*n* = 28). The participants were given general information about the experiments on arrival, and their written consent was obtained.

#### Experiment 1

The experimental procedure consisted of two periods: baseline period → condition period. Each period lasted 5 min and the participants (*n* = 50) had a 1-min rest after each period had finished. Prior to an experiment, the participants sat on a sofa while wearing headphones, and were attached to ECG transducer electrodes and an elastic chest band for 5 min to adapt them to the experimental environment. In the baseline period, their respiratory rate was controlled at 15 CPM without sound presentation. When the listening session started (condition period), the participants were presented with a 5-min drum sound sequence whose tempo was set at 80 BPM, while their respiration rate was controlled at 20 CPM. One might suspect that the change in respiratory rate would influence the HR, however, there is no effect on the HR when the respiratory rate increases from 15 CPM to 20 CPM^47^. The experimental procedures are shown in Fig. 1A.

#### Experiment 2

The experimental procedure consisted of five sessions. The participants (*n* = 18) had a 5-min rest period after each session had finished. Each session lasted for 8 min and consisted of two periods: baseline period → condition period. In the 3-min baseline period, we calculated the basal HR by averaging the HR. When the listening session started (condition period), we presented 5-min sound stimuli at a constant tempo based on the basal HR. The tempo of the sound stimuli was determined for each session by the equation described below. In the equation, x represents the rate of increase (0, 5, 10, 15 or 20) compared with the basal HR, and the basal HR represents the value calculated by averaging the HR of the first three minutes of each session (baseline period).


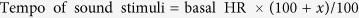


We varied the x value and defined “conditions 2–1 to 2–5”. These conditions are shown in Table 2. The respiratory rate was controlled at 20 CPM.

The five sessions all took place on one day. The condition number order is counter-balanced for each participant. The experimental procedures are shown in Fig. 2A. The equation and an example of Experiment 2 are shown in Fig. 2B.

#### Experiment 3

The experimental procedure consisted of five sessions. The participants (*n* = 28) had a 5-min rest period after each session had finished. Each session lasted for 8 min and consisted of two periods: baseline period → condition period. In the 3-min baseline period, we calculated the basal HR by averaging the HR. When the listening session started (condition period), we presented sound stimuli with a gradually increasing tempo based on the basal HR. The tempo of the sound stimuli was determined in each session by the equation given below. In the equation, x represents the rate of increase per minute (0, 1, 2, 3 or 4) and basal HR represents the value calculated by averaging the HR of the first three minutes of each session (baseline period). We set the basal HR at the initial sound tempo, which we then continuously increased.


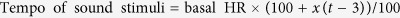


We varied the x value and defined “conditions 3–1 to 3–5”. These conditions are shown in Table 2. The respiratory rate was controlled at 20 CPM.

The five sessions all took place on one day. The condition number order was counter-balanced for each participant. The experimental procedures are shown in Fig. 3A. The equation and an example of Experiment 3 are shown in Fig. 3B.

### Data Analysis and Statistical Evaluation

In Experiment 1, time t = 0 was set as the start of each period and the HR change was evaluated by averaging the data from t = 2 to t = 5 (defined as mean HR) to exclude orienting and defense responses. In Experiment 2, time t = 0 was set as the start of each session and the HR change was evaluated by averaging the data from t = 5 to t = 8 (defined as mean HR) to exclude orienting and defense responses. For HRV analysis, the change in lnHF and the lnLF/lnHF ratio were evaluated by averaging the data from t = 5 to t = 7 and t = 6 to t = 8 to stabilize the motion artifact. In Experiment 3, time t = 0 was set as the start of each session, and the HR change was evaluated by averaging the data with a 1-min window (t = 3 to 4, t = 4 to 5, t = 5 to 6, t = 6 to 7, t = 7 to 8 for each) in the condition period (defined as mean HR). The mean HRs of the 1-min windows were defined as M1, M2, M3, M4 and M5 in 5-min condition periods, and the corresponding 1-min time regions were defined as T1, T2, T3, T4, and T5, respectively (Fig. 3C). For HRV analysis, the change in lnHF and the lnLF/lnHF ratio were evaluated by averaging the data from t = 5 to t = 7 and t = 6 to t = 8 because a 1-min window to stabilize the motion artifact. In Experiment 1, the basal HR was evaluated by averaging the data from t = 2 to t = 5 in the baseline period. In Experiments 2 and 3, the basal HR of the baseline period was evaluated by averaging the data from t = 0 to t = 3. For HRV analysis, the basal lnHF and basal lnLF/lnHF ratio of the baseline period were evaluated by averaging the data from t = 1 to t = 3 to adjust the analyzed window of the condition period.

We excluded participants from the analysis whose respiration was not synchronized with the metronome. The major reasons for this were that the participant was sleeping during the measurement or coughing too much. We excluded 2 individuals from Experiment 1, 3 individuals from Experiment 2, and 7 individuals from Experiment 3.

In Experiment 1, the respirations of 48 participants were synchronized with an electronic metronome. Since there were great differences between the basal HRs of the participants during baseline recording, we normalized the mean HR for the condition period by dividing them by the basal HR of the baseline recording. We used Pearson’s correlation method to evaluate the correlation between the ratio of change in the mean HR, and the basal HR and the basal HR itself.

In Experiment 2, the respirations of 15 participants were synchronized with an electronic metronome. We normalized the mean HR, the average lnHF and the average lnLF/lnHF ratio for each condition by dividing them by the basal HR, the basal lnHF and the basal lnLF/lnHF ratio in the baseline recording, respectively. The difference between the normalized mean HRs, the normalized lnHF and the normalized lnLF/lnHF were analyzed with a two-way repeated measures ANOVA [condition (5) x time (2)] as within-subjects factors. Then the difference between the normalized value and the baseline period was analyzed with a simple main effect test. The difference between the normalized values of the condition numbers was analyzed with Ryan’s method.

In Experiment 3, the respirations of 21 participants were synchronized with an electronic metronome. We normalized the mean HRs M1-M5 for each condition by dividing them by the basal HR in the baseline recording. The difference between the normalized mean HRs was analyzed with a two-way repeated measures ANOVA [condition (5) x time (6)] as within-subjects factors. Then the difference between the normalized value and the baseline period was analyzed with Dunnett’s method. The difference between the normalized values of the condition numbers was analyzed with Ryan’s method. The difference between the normalized lnHFs and the normalized lnLF/lnHF ratios were analyzed with a two-way repeated measures ANOVA [condition (5) x time (2)] as within-subjects factors. Then the differences between the normalized values and the baseline period were analyzed with a simple main effect test. The difference between the normalized values of the condition numbers was analyzed with Ryan’s method.

The significance was defined as *p* < 0.05. For repeated measures ANOVA, Huynh-Feldt corrections were applied when appropriate.

## Additional Information

**How to cite this article:** Watanabe, K. *et al*. Heart rate responses induced by acoustic tempo and its interaction with basal heart rate. *Sci. Rep.* **7**, 43856; doi: 10.1038/srep43856 (2017).

**Publisher's note:** Springer Nature remains neutral with regard to jurisdictional claims in published maps and institutional affiliations.

## Supplementary Material

Supplementary Tables

## Figures and Tables

**Table 1:** The percentage and SEM of normalized mean HRs of the 1-min windows in Experiment 3.

Condition number	M1	M2	M3	M4	M5
3–1	99.64 ± 0.78	99.71 ± 0.56	100.16 ± 0.66	100.60 ± 0.79	101.04 ± 0.85
3–2	101.07 ± 0.83	102.10 ± 0.92	102.25 ± 0.85	102.71 ± 0.56	101.62 ± 0.75
3–3	102.19 ± 0.66	103.90 ± 0.82	103.95 ± 0.74	105.21 ± 1.08	106.07 ± 1.00
3–4	101.41 ± 0.79	101.99 ± 0.72	102.53 ± 0.77	102.43 ± 0.69	101.79 ± 0.95
3–5	101.58 ± 0.71	102.13 ± 0.98	102.54 ± 0.77	103.97 ± 0.75	101.85 ± 0.86

Each data represents the mean HRs of the 1-min windows shown in Fig. 3C. The data represents the percentage of the basal HR in the baseline recording and SEM.

**Table 2:** The details of the experimental conditions used for Experiment 2 and 3.

Condition number	Respiratory rate (CPM)	*x* (rate of increase)
2–1	20	0
2–2		5
2–3		10
2–4		15
2–5		20
3–1	20	0
3–2		1
3–3		2
3–4		3
3–5		4

The tempo of the sound stimuli varies in each condition. The tempo is determined by the following equations for each Experiment. In Experiment 2, 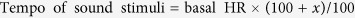. In Experiment 3, 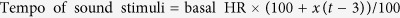. Basal HR represents the value calculated by averaging the HR of the first three minutes of each session. CPM; cycles per minute.
