# Supplementary material for: Heart rate responses induced by acoustic tempo and its interaction with basal heart rate
Source: Sci Rep. 2017 Mar 7;7:43856. doi: 10.1038/srep43856 (PMC5339732; doi:10.1038/srep43856)
Supplement: Supplementary Tables [file srep43856-s1.pdf]

Title:

Heart rate responses induced by acoustic tempo and its interaction with basal heart rate

Ken Watanabe [1*], Yuuki Ooishi [2], Makio Kashino [1,2,3]

[1]Department of Information Processing, Interdisciplinary Graduate School of Science and Engineering, Tokyo Institute of Technology, 4259 Nagatsuta-cho, Midori-ku, Yokohama, Kanagawa 226-8503, Japan.

[2]NTT Communication Science Laboratories, NTT Corporation, 3-1, Morinosato Wakamiya Atsugi, Kanagawa 243-0198, Japan.

[3]Core Research for Evolutional Science and Technology, Japan Science and Technology Agency (CREST, JST), Atsugi, Kanagawa 243-0198, Japan.

* Corresponding author.

Supplemental Table 1.

Condition number	HR (BPM)	ln HF (ln ms^2)	ln LF / lnHF
2-1	70.87±7.63 (min: 59.28, max: 83.56)	5.23±0.69 (min: 4.00, max: 6.35)	1.04±0.16 (min: 0.75, max: 1.26)
2-2	68.96±7.73 (min: 58.41, max: 85.95)	5.13±0.82 (min: 4.05, max: 6.74)	1.09±0.20 (min: 0.86, max: 1.50)
2-3	68.20±7.95 (min: 56.17, max: 83.14)	5.48±1.01 (min: 4.32, max: 7.49)	1.02±0.23 (min: 0.70, max: 1.33)
2-4	67.67±6.17 (min: 58.67, max: 78.14)	5.04±0.84 (min: 3.21, max: 6.48)	1.00±0.19 (min: 0.63, max: 1.41)
2-5	67.34±7.67 (min: 57.92, max: 85.88)	5.14±1.01 (min: 3.17, max: 6.82)	0.97±0.16 (min: 0.76, max: 1.36)

Table. S1. The basal value of HR, lnHF and lnLF/lnHF in Experiment 2.

The data represents the basal value of HR, lnHF and lnLF/lnHF in the baseline recording for each condition in Experiment 2. Each data represent the mean±SD, minimum value and maximum value for each condition shown in Table 2.

Supplemental Table 2.

Condition number	HR (BPM)	ln HF (ln ms^2)	ln LF / lnHF
3-1	72.03±8.06 (min: 57.84, max: 86.01)	4.69±0.93 (min: 2.58, max: 6.12)	1.25±0.23 (min: 0.84, max: 1.80)
3-2	71.41±7.58 (min: 61.07, max: 85.85)	4.64±0.85 (min: 2.44, max: 6.34)	1.21±0.19 (min: 0.74, max: 1.49)
3-3	69.96±7.01 (min: 61.40, max: 81.59)	4.82±0.86 (min: 2.80, max: 5.94)	1.11±0.25 (min: 0.75, max: 1.65)
3-4	69.76±8.40 (min: 57.16, max: 84.28)	4.73±1.28 (min: 2.18, max: 8.66)	1.19±0.27 (min: 0.71, max: 1.98)
3-5	69.63±8.51 (min: 57.68, max: 84.86)	4.52±0.97 (min: 2.56, max: 6.05)	1.20±0.30 (min: 0.83, max: 2.02)

Table. S2. The basal value of HR, lnHF and lnLF/lnHF in Experiment 3.

The data represents the basal value of HR, lnHF and lnLF/lnHF in the baseline recording for each condition in Experiment 3. Each data represent the mean±SD, minimum value and maximum value for each condition shown in Table 2.
